# Genomic Analysis of Multiresistant Staphylococcus capitis Associated with Neonatal Sepsis

**DOI:** 10.1128/AAC.00898-18

**Published:** 2018-10-24

**Authors:** Glen P. Carter, James E. Ussher, Anders Gonçalves Da Silva, Sarah L. Baines, Helen Heffernan, Thomas V. Riley, Roland Broadbent, Antje van der Linden, Jean Lee, Ian R. Monk, Timothy P. Stinear, Benjamin P. Howden, Deborah A. Williamson

**Affiliations:** a Department of Microbiology and Immunology, Doherty Institute for Infection and Immunity, The University of Melbourne, Melbourne, Australia; b Department of Microbiology and Immunology, University of Otago, Dunedin, New Zealand; c Southern Community Laboratories, Dunedin, New Zealand; d Microbiological Diagnostic Unit Public Health Laboratory, Department of Microbiology & Immunology, Doherty Institute, The University of Melbourne, Melbourne, Australia; e Institute of Environmental Science and Research, Wellington, New Zealand; f Department of Microbiology, PathWest Laboratory Medicine, Perth, Western Australia, Australia; g School of Medicine, University of Otago, Dunedin, New Zealand

**Keywords:** coagulase-negative staphylococci, genomics, multidrug resistance, neonates, plasmids

## Abstract

Coagulase-negative staphylococci (CoNS), such as Staphylococcus capitis, are major causes of bloodstream infections in neonatal intensive care units (NICUs). Recently, a distinct clone of S. capitis (designated S. capitis NRCS-A) has emerged as an important pathogen in NICUs internationally. Here, 122 S. capitis isolates from New Zealand (NZ) underwent whole-genome sequencing (WGS), and these data were supplemented with publicly available S. capitis sequence reads. Phylogenetic and comparative genomic analyses were performed, as were phenotypic assessments of antimicrobial resistance, biofilm formation, and plasmid segregational stability on representative isolates. A distinct lineage of S. capitis was identified in NZ associated with neonates and the NICU environment. Isolates from this lineage produced increased levels of biofilm, displayed higher levels of tolerance to chlorhexidine, and were multidrug resistant. Although similar to globally circulating NICU-associated S. capitis strains at a core-genome level, NZ NICU S. capitis isolates carried a novel stably maintained multidrug-resistant plasmid that was not present in non-NICU isolates. Neonatal blood culture isolates were indistinguishable from environmental S. capitis isolates found on fomites, such as stethoscopes and neonatal incubators, but were generally distinct from those isolates carried by NICU staff. This work implicates the NICU environment as a potential reservoir for neonatal sepsis caused by S. capitis and highlights the capacity of genomics-based tracking and surveillance to inform future hospital infection control practices aimed at containing the spread of this important neonatal pathogen.

## Abstract (precis)

Coagulase-negative staphylococci (CoNS), such as Staphylococcus capitis, are major causes of bloodstream infections in neonatal intensive care units (NICUs). Recently, a distinct clone of S. capitis (designated S. capitis NRCS-A) has emerged as an important pathogen in NICUs internationally.

## INTRODUCTION

Coagulase-negative staphylococci (CoNS) are the most common pathogens involved in nosocomial sepsis in neonatal intensive care units (NICUs) and are often associated with the presence of intravascular devices (1). Although Staphylococcus epidermidis is the most frequently isolated pathogen in CoNS bacteremia in NICUs, studies have highlighted the emergence of methicillin-resistant Staphylococcus capitis as an important opportunistic pathogen in the NICU setting (2–5). A genetically distinct clone of S. capitis, designated NRCS-A by pulse-field gel electrophoresis (PFGE), has been identified as a common cause of NICU-associated bacteremia in France and more recently in the United Kingdom, Belgium, and Australia (3, 5).

Like other regions, S. capitis has been associated with neonatal bacteremia in the NICU setting in New Zealand (NZ) and has been responsible for a sustained series of neonatal bacteremia episodes in the NICU located in Dunedin Hospital in the South Island of NZ. PFGE analysis has confirmed that the Dunedin NICU isolate is part of the NRCS-A clone of S. capitis (5). To date, however, the global population structure of S. capitis has not been comprehensively assessed using whole-genome sequencing (WGS), and the genomic relatedness of S. capitis isolates in NZ relative to those circulating in other NICUs around the world is unknown. Here, we describe the emergence and microevolution of S. capitis in NZ in the context of a globally diverse data set. Further, we identify genotypic and phenotypic factors that may contribute to the emergence and spread of S. capitis in the NICU setting and facilitate the success of S. capitis as a neonatal pathogen.

## RESULTS

### Population structure of NICU-associated S. capitis and emergence in NZ.

WGS was performed on 122 S. capitis isolates that were collected between January 2007 and July 2016 (additional information relating to isolate collection is detailed in Supplemental Methods and Data Set S1 in the supplemental material). These included S. capitis isolates obtained from neonates (*n* = 56), staff (*n* = 13), and the environment (*n* = 9) in the Dunedin NICU (see below and Data Set S1). For context, the Dunedin NICU isolates were supplemented with a collection of 12 S. capitis isolates from adult patients in Dunedin (representing 12 adult blood culture contaminants), 21 S. capitis isolates from adults in two other cities in NZ (Auckland and Christchurch), and 11 S. capitis isolates from adults in one city in Australia (Perth) (Data Set S1). In addition, 13 publicly available S. capitis genomes (available at the time of this study) were included in the genomic analysis. In total, 135 S. capitis isolates were included in phylogenetic analyses.

A complete S. capitis reference genome (NZ-SC16875) was generated using PacBio single-molecule real-time (SMRT) technology. NZ-SC16875 was one of the earliest isolates collected in this study, being obtained in 2011 from a neonate in Dunedin with bacteremia. Sequence reads were mapped to NZ-SC16875, and 75,177 single nucleotide polymorphisms (SNPs) were present within the core genome and were used to construct a maximum likelihood (ML) phylogeny following filtering for recombination. There was extensive recombination among the study isolates (see Fig. S1 in the supplemental material), with recombination most evident within the ancestral branches leading to the main Bayesian Analysis of Population Structure (BAPS) groups, rather than within any of the specific groups. With respect to the NICU-associated BAPS 3 (BP3) group, recombination was inferred across large regions of the chromosome, predominantly within the first ∼600 kb and the last ∼1 Mb of the genome. These events generally mapped to the ancestral branch leading up to the BP3 group. This suggests, therefore, that the major lineages of S. capitis may have arisen following extensive recombination events.

**FIG 1:**
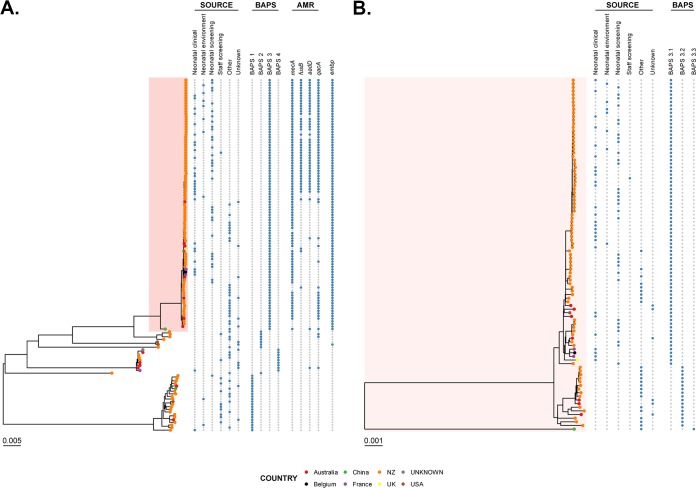
Maximum likelihood (ML) trees demonstrating the population structure of Staphylococcus capitis. ML trees were inferred from core-genome single nucleotide polymorphisms (SNPs) and rooted using the minimal ancestor deviation method (29). (A) ML tree of all 135 included S. capitis isolates, with metadata indicating source, highest level BAPS grouping, and presence/absence of antimicrobial resistance (AMR) genes (*mecA* and plasmid-carried *fusB*, *aadD*, and *qacA*) and *embp*. (B) ML tree of isolates classified as part of BAPS group 3, with metadata indicating source and BAPS grouping of isolates within BAPS group 3 (indicated in panel A).

The recombination-filtered population structure was assessed using a Bayesian hierarchical clustering approach, which identified four distinct groups (BAPS groups). NICU-associated isolates (clinical, screening, and environmental) were found predominantly within BAPS group 3 (BP3), while BAPS group 1, 2, and 4 (BP1, BP2, and BP4, respectively) isolates were most often associated with non-NICU samples (Fig. 1A). Importantly, with one exception (a staff member identified in 2014 as part of screening), isolates obtained from Dunedin NICU staff clustered predominantly within BP1 rather than BP3 (Fig. 1A), suggesting that the Dunedin NICU staff who were screened did not form a major reservoir for S. capitis NICU sepsis.

### The NICU environment is a potential reservoir for neonatal sepsis caused by multidrug-resistant BP3 lineage S. capitis.

To further understand the population structure and clonal dissemination of the BP3 group, a second phylogenetic analysis was performed on BP3 isolates only using the local NZ-SC16875 reference genome (Fig. 1B). Within BP3, NICU-associated isolates from NZ clustered together in a distinct clade, designated BP3.1 (Fig. 1B). Importantly, many of the NZ BP3.1 lineage isolates obtained from neonates (both blood culture isolates and screening specimens) were indistinguishable at a core-genome level from those isolates collected from the NICU environment over a 12-month period. This included isolates collected from stethoscopes and the internal surface of neonatal incubators, suggesting that the NICU environment and associated fomites might act as a possible reservoir of S. capitis neonatal infections.

In addition to NICU-associated isolates from Dunedin, the BP3.1 lineage contained isolates that were temporally and geographically diverse. These included isolates from adults from other cities in NZ and five international isolates from Australia, Belgium, France, the United States, and the UK which were previously associated with neonatal sepsis (Fig. 1B). However, these isolates showed marked variation in their accessory genome compared to NZ NICU isolates and did not harbor the NZ NICU-associated pSC16875 plasmid (see below).

### Complete genome sequence of multidrug-resistant NICU-associated S. capitis NZ-SC16875.

**FIG 2:**
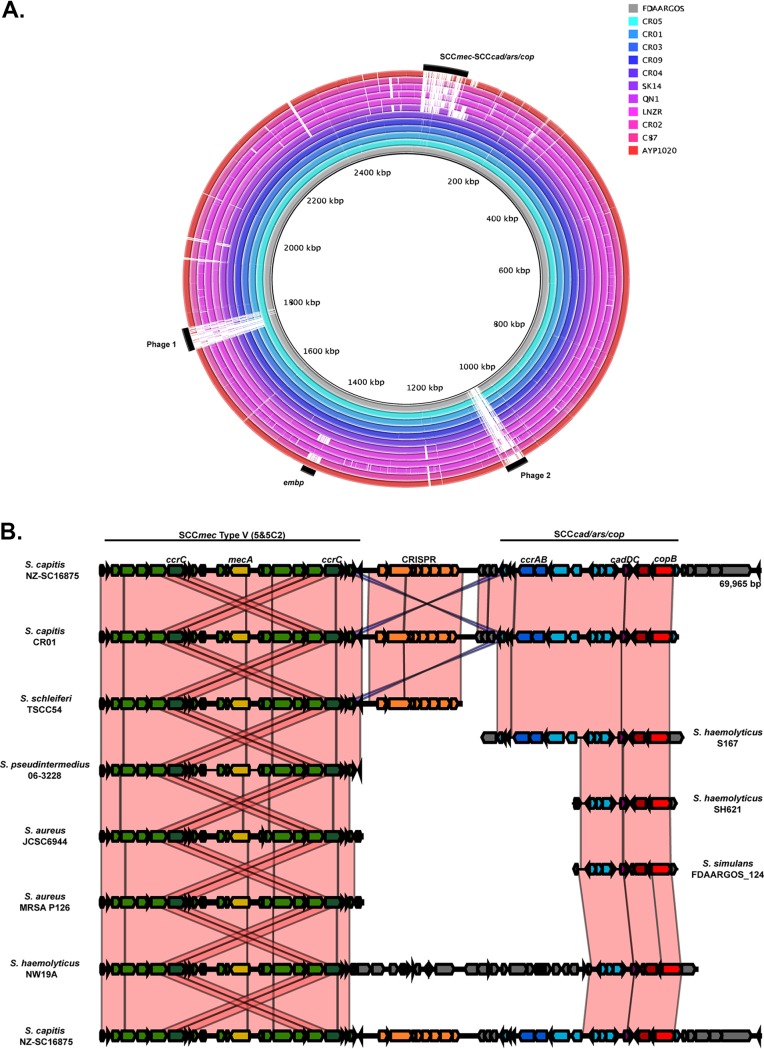
(A) Comparison of representative S. capitis chromosome sequences relative to S. capitis NZ-SC16875, illustrating a high level of conservation among the international NICU-associated S. capitis BP3 isolates (inner six rings). Variable regions are annotated in the outer ring. These regions include two putative bacteriophages (phage 1 and phage 2), the SCC*cad*-SCC*ars*-SCC*cop* region, and the *embp* gene (B) Schematic diagram illustrating the genetic organization of the composite SCC*cad*-SCC*ars*-SCC*cop* element in BP3 lineage S. capitis isolates NZ-SC16875 and CR01. Also included are the SCC*mec* V elements of S. schleiferi TSCC54, S. pseudintermedius 06-3228, S. aureus JCSC6944, and MRSA P126, and S. haemolyticus NW19A, as well as the SCC*cad*-SCC*ars*-SCC*cop* regions of S. haemolyticus S167 and SH621 and S. simulans FDAARGOS_124 for comparison. Green and yellow arrows indicate sequences present within SCC*mec* V regions; orange arrows are CRISPR-associated genes; and blue, purple, and red arrows indicate genes within SCC*cad*-SCC*ars*-SCC*cop* regions. The direction of the arrows indicates the direction of transcription for open reading frames. Only coding sequences of >200 bp are shown. Shaded areas represent regions that share >99% nucleotide sequence identity (pink denotes the same orientation, and blue denotes the reverse orientation). Note that the S. capitis NZ-SC16875 region is shown at the top and bottom of the figure for ease of comparison.

The genome of NZ-SC16875 comprised a circular chromosome of 2,516,968 bp and a single circular plasmid (termed pSC16875) of 28,248 bp. A nucleotide comparison of the chromosomes of representative draft and complete S. capitis genome sequences in GenBank (http://www.ncbi.nlm.nih.gov/GenBank/) relative to NZ-SC16875 is illustrated in Fig. 2A. Visual assessment of the alignment revealed high conservation of the genome between members of the species, with variable regions associated predominantly with mobile genetic elements (MGEs) (Fig. 2A). These MGEs included two presumptive prophages and a 60.9-kb composite staphylococcal cassette chromosome (SCC) that displayed 99.9% nucleotide identity to the composite SCC*mec*-SCC*cad*-SCC*ars*-SCC*cop* element previously described in NCRS-A CR01, isolated from the blood culture of an infant diagnosed with sepsis in 2007 in France (GenBank accession no. KF049201) (6). This SCC*mec*-SCC*cad*-SCC*ars*-SCC*cop* element was also present in five other publicly available NICU-associated S. capitis genomes from different countries (Fig. 2A). Previous work has suggested that this element is a hybrid between a 38.8-kb SCC*mec* V region of a sequence type 398 (ST398) methicillin-resistant Staphylococcus aureus (MRSA) isolate and a 22.2-kb SCC*cad*-SCC*ars*-SCC*cop* region from staphylococcal species, such as S. epidermidis and S. haemolyticus (6). However, a BLAST search (http://blast.ncbi.nlm.nih.gov) of both the SCC*mec* region and the SCC*cad*-SCC*ars*-SCC*cop* region against publicly available genomic data revealed a high level of identity to SCC elements in numerous other staphylococcal species, including Staphylococcus schleiferi and Staphylococcus pseudintermedius (Fig. 2B). The conservation of the large *SCCmec*-SCC*cad*-SCC*ars*-SCC*cop* element across NICU-associated S. capitis isolates from separate geographic origins is suggestive of widespread clonal dissemination of a distinct S. capitis lineage rather than multiple independent introductions of this element into locally circulating S. capitis isolates. This hypothesis is in keeping with the findings of Butin et al., who recently used PFGE analysis on multiple international S. capitis isolates to highlight the global dissemination of the NRCS-A S. capitis clone (3).

NZ-SC16875 also harbored genes associated with biofilm formation, including the *icaADBC* operon, which produces polysaccharide intercellular adhesion (PIA), essential for biofilm formation in S. epidermidis (7). In all included genomes, the *icaADBC* region displayed >99% nucleotide identity to a previously sequenced S. capitis *icaADBC* operon (GenBank accession no. JF930147) (8). In addition, NZ-SC16875 contained a 25.5-kb gene (*embp*) encoding the large extracellular matrix-binding protein (Embp) (9). The *embp* gene was also present in 10 publicly available genomes (Fig. 2A), five of which were obtained from NICU-associated S. capitis isolates. Notably, the *icaADBC* operon was present in S. capitis isolates from all BAPS groups, whereas *embp* was almost exclusively present in the BP3 group (Fig. 1A). This observation is in keeping with the findings of Simões et al., who recently identified the *ebh* gene (an alternative name for *embp*) as a potential virulence factor in a small number of other recently sequenced S. capitis isolates (10).

### Characterization of the NZ-associated pSC16875 multidrug resistance plasmid.

A total of 42 NZ NICU isolates (clinical and environmental) harbored a plasmid (pSC16875) which carried antimicrobial resistance determinants, namely, *blaZ*, *fusB*, and *aadD* (Fig. 1B). In keeping with these genotypic findings, isolates containing pSC16875 were phenotypically resistant to amoxicillin, fusidic acid, and aminoglycosides. In addition, pSC16875 also harbored the *qacA* gene, associated with reduced susceptibility to chlorhexidine (see below). Comparison with the other publicly available closed NICU-associated S. capitis plasmid (CRO1; GenBank accession no. LN866850) revealed minimal nucleotide identity with pSC16875 (Fig. S1), suggesting independent, possibly local acquisition of this novel plasmid, or the expansion of a successful clone following its introduction into NZ. All isolates within the BP3 NICU-associated clade (in addition to other global NICU-associated S. capitis isolates) harbored the chromosomal *aac(6′)-aph(2′)* gene and were phenotypically resistant to gentamicin.

### Phenotypic properties of NZ NICU-associated S. capitis.

The results from our genomic analyses were used to inform the assessment of phenotypic traits that may have contributed to the success of the BP3 S. capitis NICU lineage in NZ. First, given the finding of the *embp* gene predominantly in BP3 isolates (Fig. 1A), we compared biofilm formation in representative S. capitis BP3 isolates with representative isolates from the S. capitis BP1, BP2, and BP4 groups. Unlike BP3 and BP4 isolates, BP1 and BP2 isolates did not produce detectable biofilm (Fig. S2A). BP3 isolates produced significantly more biofilm than isolates from BP4 (mean ± standard error of the mean [SEM] optical density at 590 nm [OD_590_], 2.775 ± 0.01 versus 0.795 ± 0.16, respectively; *P* < 0.001) (Fig. S2A), suggesting a possible advantage for these isolates in the NICU setting, as well as more broadly.

Second, to determine the stability of pSC16875 with and without the selective pressure of antimicrobials, plasmid segregational stability was assessed. pSC16875 was stably maintained under the conditions tested, with no plasmid loss observed after 1 week of serial passage, regardless of exposure to antimicrobials (Fig. S2B). This demonstrates that the multiresistant phenotype conferred by pSC16875 carriage can be maintained in this clone even in the absence of direct antimicrobial selection (Fig. S2B).

Third, to investigate the association between the carriage of *qacA* of pSC16875 and chlorhexidine tolerance, chlorhexidine susceptibility testing was performed. Isolates that harbored *qacA* (on pSC16875) had significantly higher chlorhexidine MICs and minimum bactericidal concentrations (MBCs) than those of isolates without *qacA* (median MIC, 4 mg/liter versus 1 mg/liter, respectively, *P* < 0.001; median MBC, 4 mg/liter versus 1.5 mg/liter, respectively, *P* < 0.0001) (Fig. S2C). In addition, isolates that harbored *fusB* on pSC16875 also had significantly higher fusidic acid MICs than those without *fusB* (Fig. S2D).

Finally, none of the tested isolates displayed heterogeneous intermediate vancomycin resistance, based on criteria defined for S. aureus, since there are no criteria for S. capitis or CoNS generally.

## DISCUSSION

Taken together, our analyses support the worldwide dissemination of a distinct S. capitis NICU-associated clone, NRCS-A, previously reported by Butin et al., which we believe has then undergone successful local adaptation in the NZ setting, predominantly through the acquisition of a multidrug-resistant plasmid (5). The global dissemination of this health care-associated clone has parallels with the emergence and local adaptation of other staphylococcal clones that are ecologically associated with the hospital environment. For example, epidemic MRSA-15 (EMRSA-15) belonging to sequence type 22 (ST22), emerged as an important cause of nosocomial bacteremia in the UK in the 1990s. It has previously been suggested that subsequent global spread of EMRSA-15 may have been facilitated by the movement of health care workers across the globe (11). In our study, the mode of transmission of S. capitis could not be determined, although our finding that NICU-associated isolates from multiple continents were highly related at a core-genome level (in contrast to the diverse population structure of S. capitis from adults) suggests global dissemination, possibly via the intercontinental movement of health care workers, in keeping with the findings of others (5). However, staff screening undertaken as part of infection control measures in Dunedin demonstrated that apart from one isolate, staff isolates were a different lineage of S. capitis (BP1) rather than the BP3 neonatal lineage, although it is possible that staff colonization is transient or is present in only a small number of individuals. However, given our finding of highly related S. capitis isolates from neonates and the NICU environment, it is also possible that the environment is an important reservoir for this multidrug-resistant clone. As such, an alternative possibility for the global dissemination of this pathogen is through point-source contamination of equipment or products used in the NICU setting. This theory has parallels with the international clonal dissemination of another pathogen, Mycobacterium chimaera, which has spread across the globe through contaminated heater-cooler devices used in cardiac surgery (12). However, seven of eight positive environmental samples were from stethoscopes, while the other positive site was an incubator. These items were in contact with the skin of colonized babies; therefore, it is not possible to determine if the environment is the reservoir of S. capitis or if the colonized neonates have contaminated their environment. Future studies should investigate the potential reservoirs and vehicles that have permitted the global dissemination of this strain.

Another key finding from our study was the presence of the multidrug-resistant plasmid pSC16875 in NZ S. capitis NICU-associated isolates. The observation that pSC16875 was only found in NZ NICU isolates suggests possible acquisition in NZ, perhaps under selection pressure from local antimicrobial and antiseptic use, although we acknowledge that sample bias might be a confounding factor in this observation. Of note, NZ NICU isolates were commonly resistant to fusidic acid, mediated by plasmid-borne *fusB*. Fusidic acid is used extensively in NZ (13), and consequently, NZ has one of the highest rates of fusidic acid resistance among S. aureus bacteria in the developed world (14). Under selection pressure from fusidic acid, dominant clones of ST1 methicillin-susceptible S. aureus and ST5 MRSA have emerged in the NZ setting (14, 15). Like S. aureus, it is plausible that widespread use of fusidic acid in NZ has contributed to the local selection of fusidic acid-resistant S. capitis. pSC16875 also harbored *qacA*, which encodes a multidrug resistance efflux pump that can extrude chlorhexidine (16). The presence of *qacA* has been variably correlated with increased chlorhexidine MICs and MBCs in S. aureus (16), but chlorhexidine susceptibility has not previously been systematically compared in *qacA*-positive and *qacA*-negative S. capitis isolates. Our finding that S. capitis isolates with *qacA* had significantly higher chlorhexidine tolerance is of concern in the NICU setting, where chlorhexidine is often used extensively for staff and environmental decontamination. Given the plasmid colocation of *qacA* and other resistance determinants, future work should assess the impact of chlorhexidine usage on coselection for multiresistant S. capitis isolates. Unlike previous studies assessing antimicrobial resistance in S. capitis (2, 3, 17), no resistance or heterogeneous vancomycin-intermediate resistance was observed in NICU-associated S. capitis isolates from NZ. A previous study suggested that heterogeneous vancomycin-intermediate resistance in S. capitis NRCS-A isolates may be driven by local use of vancomycin (17), and it is possible that low use of vancomycin in the NICU setting in NZ, which occurs only occasionally after a CoNS isolate is cultured, has mitigated the clonal spread of heteroresistant S. capitis.

In addition to antimicrobial resistance, biofilm formation has also been suggested to be an important virulence characteristic of S. capitis in the NICU environment (8). In keeping with this suggestion, we observed significantly more biofilm formation in NICU-associated BP3 S. capitis isolates than in other S. capitis isolates. Interestingly, all study isolates had identical *icaADBC* operons, but S. capitis BP3 isolates contained the *embp* gene, which was absent in most non-BP3 strains. The product of *embp* (Embp) was recently demonstrated to be a multifunctional cell surface fibronectin binding protein involved in attachment to host extracellular matrix, biofilm accumulation, and evasion of host phagocytic responses in S. epidermidis (9). In S. aureus, the giant protein Ebh (encoded by *ebh*) is a homologue of Embp, and mutations in *ebh* are associated with increased susceptibility to complement-mediated killing, in addition to attenuated virulence in mice (18). It is therefore plausible that Embp production confers a selective advantage to S. capitis in the NICU setting. Future work, including mutagenesis studies, should be conducted to test this hypothesis.

In summary, our genotypic and phenotypic analyses of multidrug-resistant S. capitis strains provide valuable insights into factors contributing to the unprecedented global emergence of S. capitis in the NICU setting and furthermore suggest that the NICU environment might act as a reservoir for neonatal sepsis caused by this important pathogen. Importantly, compared to previous studies using PFGE alone, the higher resolution afforded by WGS in our study has enabled important differences in the accessory genomes between NICU-associated S. capitis strains across different geographic regions to be uncovered, and it has allowed us to explore phenotypic traits that may provide an advantage, particularly in the local NZ NICU environment. Our phylogenomic framework will provide a valuable foundation for future studies investigating the dissemination, pathogenicity, and adaptation of this important neonatal pathogen.

## MATERIALS AND METHODS

### Epidemiological context of NICU-associated S. capitis in NZ.

New Zealand is an island nation in the South West Pacific, with a population of approximately 4.4 million. Located in Dunedin (the second largest city in the South Island of NZ), Dunedin Hospital serves a catchment population of approximately 320,000 residents. Within Dunedin Hospital, there is a 16-bed NICU providing tertiary-level neonatal care. Between January 2007 and July 2016, S. capitis was identified in 40 of 127 (31.5%) CoNS blood culture isolates in the Dunedin Hospital NICU.

From 2010, infection control measures within the NICU were intensified to include staff undertaking a 2-min scrub with chlorhexidine on entry to the NICU, staff cleaning their arms to the elbows with an alcohol-based hand sanitizer (especially when placing arms in an incubator), and daily cleaning of ports on incubators and plastic exterior catches with benzalkonium chloride (Viraclean) wipes. In September 2013, a neonatal screening program was initiated, with axillary swabs collected from all new admissions and thereafter weekly from neonates who had previously tested negative. During the period of December 2013 to March 2015, 214 neonates were screened, and 61 (28.5%) neonates became colonized. In December 2013, the NICU moved to a new facility, with extensive cleaning of all equipment conducted prior to the move. Despite these measures, ongoing blood culture isolation of S. capitis and neonatal colonization episodes continued to occur. Therefore, in July and August 2014, voluntary anonymous screening of NICU staff was undertaken to investigate potential staff reservoirs. Briefly, staff were asked to swab their external ear (both inside the pinna and behind the ear) and, following hand washing with soap to remove transient skin microbiota, to make a plate impression of their thumb on a blood agar plate. As part of this staff screening program, a total of 41 isolates of S. capitis were identified.

In addition, between September and December 2013, a total of 40 environmental samples were collected. Environmental surfaces were swabbed; the swab was premoistened for dry surfaces, and swabs were cultured on blood agar at 36°C in 5% CO_2_ for 48 h. Samples were collected from stethoscopes (*n* = 13), incubators (*n* = 9), milk warming bottles (*n* = 5), breast pumps (*n* = 3), sinks (*n* = 3), ultrasound gel (*n* = 2), weighing scales (*n* = 2), and other sources (*n* = 3). Of the 40 samples, 8 (20%) samples grew the NICU-endemic S. capitis strain, with 7 from 9 stethoscope samples (78%) and 1 from 9 incubator samples (11%). Two samples from the ear pieces of stethoscopes grew unrelated S. capitis strains.

From the above-described epidemiological investigations, a total of 21 blood culture isolates (from 2007 to 2014), 34 isolates from neonatal axillary swabs or other nonsterile site neonatal clinical specimens, 9 environmental isolates from the NICU, and 13 isolates from the staff screening (3 from the thumb imprint and 10 from the pinna) were selected for further genotypic and phenotypic analyses (Table S1); where more isolates were available, isolates were selected for sequencing to ensure coverage over time and minor differences in PFGE patterns.

### Bacterial isolates and susceptibility testing.

Initial identification of S. capitis isolates was performed using matrix-assisted laser desorption ionization–time of flight (MALDI-TOF) mass spectrometry (Bruker Daltonics, USA), and urease and maltose fermentation tests were used to differentiate between S. capitis subsp. urealyticus and S. capitis subsp. capitis. Antimicrobial susceptibility testing was performed using the Vitek 2 system (bioMérieux, Marcy l'Etoile, France), or broth microdilution for fusidic acid, and was interpreted according to CLSI (19) or EUCAST guidelines (www.eucast.org). To identify heterogeneous vancomycin-intermediate resistance, all strains underwent testing using the Etest macromethod, using previously described methods and cutoffs for screening S. aureus (20). The vancomycin-susceptible S. aureus strain ATCC 29213 was used as a negative control, and the heterogeneous vancomycin-intermediate-resistant S. aureus strain Mu3 was used as a positive control (21). Chlorhexidine phenotypic susceptibility testing was performed in triplicate using standard CLSI broth microdilution methods to determine chlorhexidine MICs and minimum bactericidal concentrations (MBCs) (see Supplemental Methods in the supplemental material) (22, 23).

### Genomic and phylogenetic analysis.

Genomic DNA extraction, library preparation, and whole-genome sequencing on a NextSeq 500 platform (Illumina, Inc.), using 2 × 150-bp chemistry, was performed as previously described (24). One isolate (designated NZ-SC16875) underwent genomic DNA extraction with the GenElute bacterial genomic DNA kit (Sigma-Aldrich), followed by sequencing on the RSII (Pacific Biosciences) with P6-C4 chemistry according to the 20-kb template preparation using the BluePippin size selection system protocol (Pacific Biosciences). Sequences were initially analyzed using the Nullarbor pipeline (T. Seeman; https://github.com/tseemann/nullarbor). Phage regions were detected using Phaster (25). Maximum likelihood trees were inferred using IQ-TREE (26). Recombination was detected using Gubbins (version 2.2.0-1) (27), and population structure was investigated using hierarchical Bayesian analysis with hierBAPS (28). Additional information on bioinformatic analyses is provided in the Supplemental Methods.

### Biofilm assessment and assessment of plasmid segregational stability.

A quantitative determination of biofilm production was performed using a 96-well microtiter plate assay (see Supplemental Methods) on a representative sampling of isolates from each S. capitis BP group. Where possible, every isolate within BP groups was included; however, for the larger BP groups, representative isolates were selected that provided the greatest coverage of strain variation within the BP group. To assess plasmid stability, triplicate cultures of each test strain were grown overnight in brain heart infusion (BHI) broth, with appropriate antimicrobial selection for plasmid maintenance, followed by five daily passages under either selective or nonselective conditions. The proportion of plasmid-free cells was determined after each passage (see Supplemental Methods).

### Ethical approval.

All samples were collected as part of normal clinical care or as part of an infection prevention and control investigation. Access to clinical data for this study was approved by the University of Otago Human Ethics Committee (Health) (project HD16/050).

### Data availability.

All bacterial isolates used in this study are available upon request, and all sequence data generated have been deposited in the European Nucleotide Archive (ENA) under study accession no. PRJNA398301.

## Supplementary Material

Supplemental file 1

Supplemental file 2
