# Supplementary material for: Genomic Analysis of Multiresistant Staphylococcus capitis Associated with Neonatal Sepsis
Source: Antimicrob Agents Chemother. 2018 Oct 24;62(11):e00898-18. doi: 10.1128/AAC.00898-18 (PMC6201123; doi:10.1128/AAC.00898-18)

SUPPLEMENTARY METHODS

Chlorhexidine phenotypic susceptibility: A two-fold dilution series (to give final concentrations of 32 to 0.5 mg/L) of chlorhexidine digluconate (Sigma 20% aqueous solution) was inoculated in 100 μL volumes in a 96-well plate, along with a 100 μL suspension of *Staphylococcus capitis* from an overnight blood agar culture adjusted to give a final inoculum of 5 x 10^5 cfu/mL. The MIC was defined as the lowest chlorhexidine concentration that inhibited visible growth after 24 h incubation. To determine the MBC, 10 μL from each well showing no visible growth was plated on to non-selective Mueller–Hinton agar and the MBC defined as the lowest concentration from which no colonies grew after 48 h incubation. All MIC and MBC testing was performed in triplicate.

Bioinformatic analysis: Reads were trimmed to remove adaptor sequences and low-quality bases with Trimmomatic (1). Kraken (v0.10.5-beta, (2)) was then used to assess for contamination (using the default database), and confirm isolate species. We used snippy v3.2 (https://www.github.com/tseemann/snippy) to automate core SNP discovery, using default settings. Reads were aligned to an annotated reference genome (NZ-SC16875, detailed below) using BWA MEM (v 0.7.15-r1140, (3)). Samples with at least 45x depth of coverage and 85% genome coverage were retained for analysis. Single nucleotide polymorphisms (SNPs) were identified using FreeBayes (v1.0.2, (4)) under a haploid model, with a minimum depth of coverage of 10x and allelic frequency of 0.9 required to confidently call a SNP. SNPs were annotated using snpEff (5). The reference was annotated using Prokka (6). Phage regions were detected with Phaster (7), and were masked in full consensus alignment using bedtools (v 2.26.0) (8).

Population structure: Blocks of recombination were detected using Gubbins (v.2.2.0-1, (9)), with the phage-masked core full alignment provided as input. This analysis was run for 20 iterations. Core SNPs were identified from the final recombination-masked fasta file using snp-sites (v2.3.1) (10). The final alignment was used to produce a maximum likelihood tree in IQ-TREE (v.1.4.3, (11)), with the best-fitting model of nucleotide substitution chosen based on the lowest Bayesian Information Criterion. Confidence in the phylogeny was assessed via the ultra-fast bootstrap approximation with 10,000 iterations (12). Maximum likelihood trees were rooted using the minimum ancestor deviation algorithm (13). Population structure was

investigated using hierarchical Bayesian analysis with hierBAPS (14), with a concatenated alignment of core SNPs as input and allowing for up to 20 clusters with a maximum of 10 possible levels (i.e., subgroups within the clusters). Using TempEst (15) no temporal signal was detected based on the correlation between root-to-tip distances and isolation dates for all tips in the phylogeny; therefore, we did not proceed with dating the phylogeny.

Biofilm assessment: Overnight cultures of *S. capitis* grown in tryptic soy broth (TSB) were diluted 1 in 100 in TSB broth supplemented with 4% (w/v) NaCl, and 100 μL aliquots were subsequently grown in a 96-well microtitre tray (Corning Inc, USA). The tray was incubated statically overnight at 37 °C. Each well was then washed four times with 200 μL of phosphate buffer saline (PBS) and stained with 150 μL of 0.1% (v/v) crystal violet for 15 min. The crystal violet solution was then removed and the wells washed an additional three times with PBS before being allowed to air dry overnight. Biofilm-associated crystal violet was solubilized using 100% ethanol and the OD_{590} determined using an Ensight multimode plate reader (PerkinElmer).

Plasmid segregational stability: Each culture was grown overnight in 10 mL brain heart infusion (BHI) broth (30 mL bottle), was diluted to 0.5 McFarland in BHI broth, then diluted 1 in 100 into either non-selective BHI broths or BHI broth containing 8 mg/L fusidic acid or 2 mg/L chlorhexidine, performed in triplicate. Five consecutive overnight serial passages of each culture were performed in either non-selective broth or broth containing 8 mg/L fusidic acid or 2 mg/L chlorhexidine. At each 24 h time point, aliquots were removed from each culture, serially diluted in PBS and spread onto non-selective BHI agar plates and incubated overnight. Subsequently, 100 colonies from each culture were cross patched onto non-selective BHI agar and BHI agar supplemented with 8 mg/L fusidic acid. Plasmid segregational stability was calculated based on the proportion of colonies that were susceptible to fusidic acid compared to the number of colonies that had grown on non-selective agar.

REFERENCES

1. **Bolger AM, Lohse M, Usadel B.** 2014. Trimmomatic: a flexible trimmer for Illumina sequence data. Bioinformatics **30:**2114-2120.
2. **Wood D, Salzberg, SL.** 2014. Kraken: ultrafast metagenomic sequence classification using exact alignments. Genome Biol **15**.

3. **Li H.** 2013. Aligning sequence reads, clone sequences and assembly contigs with BWA-MEM. arXiv:1303.3997v1302.
4. **Garrison E, Marth, G.** 2012. Haplotype-based variant detection from short-read sequencing. arXiv**:**1207.3907v1202.
5. **Cingolani P, Platts A, Wang le L, Coon M, Nguyen T, Wang L, Land SJ, Lu X, Ruden DM.** 2012. A program for annotating and predicting the effects of single nucleotide polymorphisms, SnpEff: SNPs in the genome of Drosophila melanogaster strain w1118; iso-2; iso-3. Fly (Austin) **6:**80-92.
6. **Seemann T.** 2014. Prokka: rapid prokaryotic genome annotation. Bioinformatics **30:**2068-2069.
7. **Arndt D, Grant JR, Marcu A, Sajed T, Pon A, Liang Y, Wishart DS.** 2016. PHASTER: a better, faster version of the PHAST phage search tool. Nucleic Acids Res **44:**W16-21.
8. **Quinlan AR, Hall IM.** 2010. BEDTools: a flexible suite of utilities for comparing genomic features. Bioinformatics **26:**841-842.
9. **Croucher NJ, Page AJ, Connor TR, Delaney AJ, Keane JA, Bentley SD, Parkhill J, Harris SR.** 2015. Rapid phylogenetic analysis of large samples of recombinant bacterial whole genome sequences using Gubbins. Nucleic Acids Res **43:**e15.
10. **Page AJ, Taylor B, Delaney AJ, Soares J, Seemann T, Keane JA, Harris SR.** 2016. SNP-sites: rapid efficient extraction of SNPs from multi-FASTA alignments. Microb Genom **2:**e000056.
11. **Nguyen LT, Schmidt HA, von Haeseler A, Minh BQ.** 2015. IQ-TREE: a fast and effective stochastic algorithm for estimating maximum-likelihood phylogenies. Mol Biol Evol **32:**268-274.
12. **Minh BQ, Nguyen MA, von Haeseler A.** 2013. Ultrafast approximation for phylogenetic bootstrap. Mol Biol Evol **30:**1188-1195.
13. **Tria FDK, Landan, G. & Dagan, T.** 2017. Phylogenetic rooting using minimal ancestor deviation. Nat Ecol Evol **1, 0193**
14. **Cheng L, Connor TR, Siren J, Aanensen DM, Corander J.** 2013. Hierarchical and spatially explicit clustering of DNA sequences with BAPS software. Mol Biol Evol **30:**1224-1228.
15. **Rambaut L, de Carvalho & Pybus** 2016. Exploring the temporal structure of heterochronous sequences using TempEst. Virus Evolution doi:DOI: http://dx.doi.org/10.1093/ve/vew007.
16. **Hadfield J, Croucher NJ, Goater RJ, Abudahab K, Aanensen DM, Harris SR.** 2018. Phandango: an interactive viewer for bacterial population genomics. Bioinformatics **34:**292-293.

Supplementary Figure 1. Recombination blocks identified in *S. capitis* isolates.

Gubbins (9) was using to identify recombination blocks, using the core full alignment as input, with 20 iterations. Recombination blocks were visualized with Phandango (16). The recombination-adjusted maximum likelihood tree is shown, with corresponding BAPS clusters (BP1-blue; BP2-red; BP3-purple; BP4-yellow). Recombination blocks are ordered based on their position in the NZ-SC16875 reference genome (shown along the top). Blocks

detected in >1 isolate show in red, while blocks affecting a single isolate are indicated in blue.

Supplementary Figure 2. Nucleotide comparison of the ~26 kb plasmid from *Staphylococcus capitis* CR01 (Genbank Accession LN866850) and pSC16875, demonstrating limited nucleotide homology. Coding regions are represented by arrows, and nucleotide homology is represented by blue or red (inverted) regions. IS*431mec*-like transposase elements are identified by *.

Supplementary Figure 3: Independent acquisition of plasmid pSC16875 has conferred additional antimicrobial resistance in the New Zealand NICU-associated BP3 clone. **Panel A:** Comparison of biofilm formation by representative strains of each *S. capitis* BAPS group. For BP1 and BP3 (n=10), while for BP2 and BP4 (n=6). Quintuplicate technical replicates were performed for each isolate. Error bars represent the standard error of the mean (SEM) of biological replicates. **Panel B:** Segregational stability of plasmid pSC16875 in *S. capitis* NZ-SC16875. Cultures of *S. capitis* NZ-SC16875 were serially passaged in either non-selective broth (red), or in broth supplemented with 8 mg/L fusidic acid (green) or with 2 mg/L chlorhexidine (black). Plasmid loss was determined every 24 h for 5 days and then at 7 days as indicated. The mean of triplicate independent cultures grown under each test condition are shown. Note that error bars are absent since the plasmid was segregationally stable in each replicate under all conditions tested. The green and black lines have been displaced downwards for visualization purposes. **Panel C:** Chlorhexidine broth micro-dilution minimum inhibitory concentration (MIC) assays (pSC16875-negative isolates (n=10) and pSC16875-positive isolates (n=10)); **Panel D:** fusidic acid broth micro-dilution MIC assays

(pSC16875-positive isolates (n=42) and pSC16875-negative isolates (n=45)). In both graphs, boxes represent the range of MIC values and the central line denotes the median.

Supplementary Data: Metadata associated with *S. capitis* study isolates. Includes isolate collection information, antimicrobial resistance gene carriage information and whole genome sequence accession numbers.

Supplementary Figure 1.

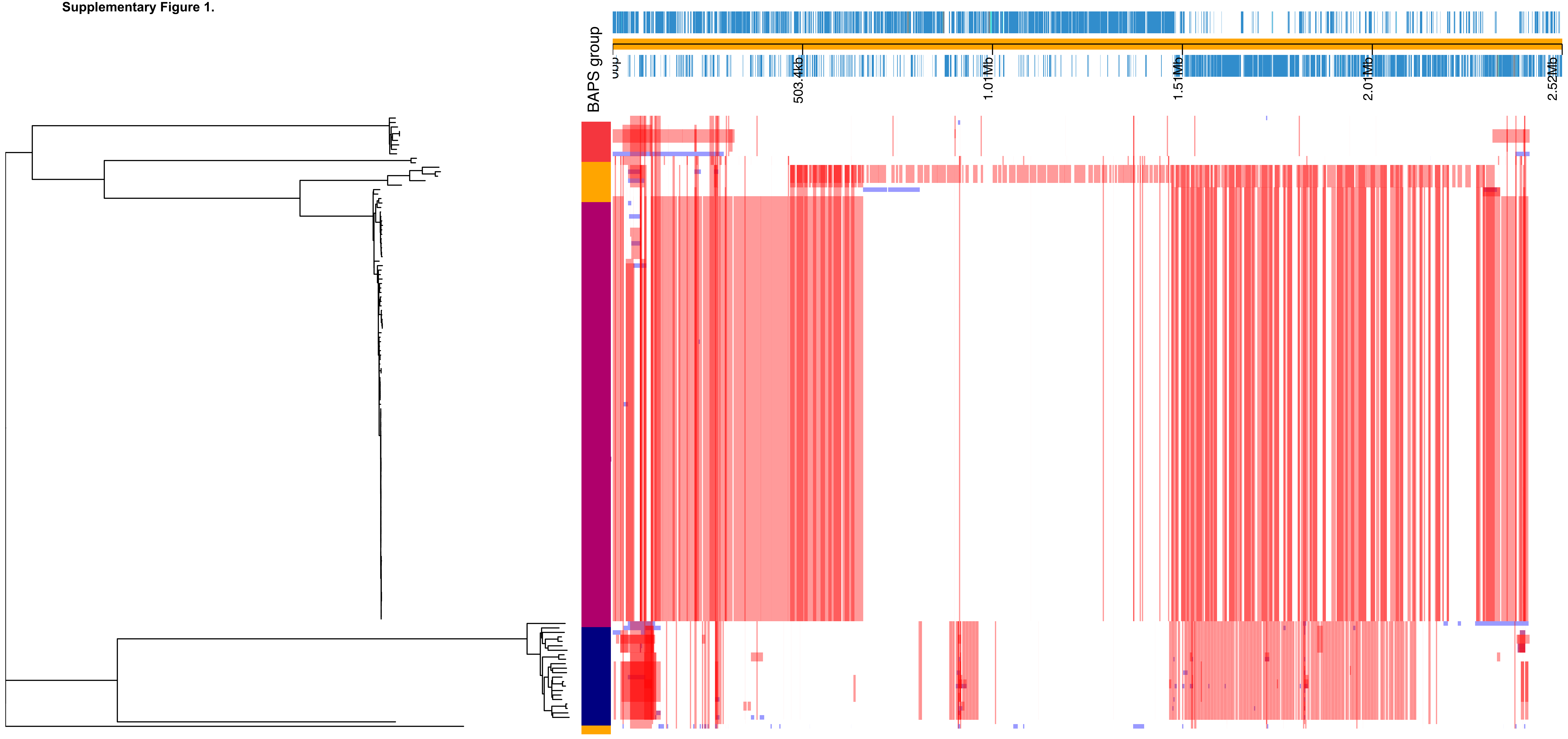

Supplementary Figure 2.

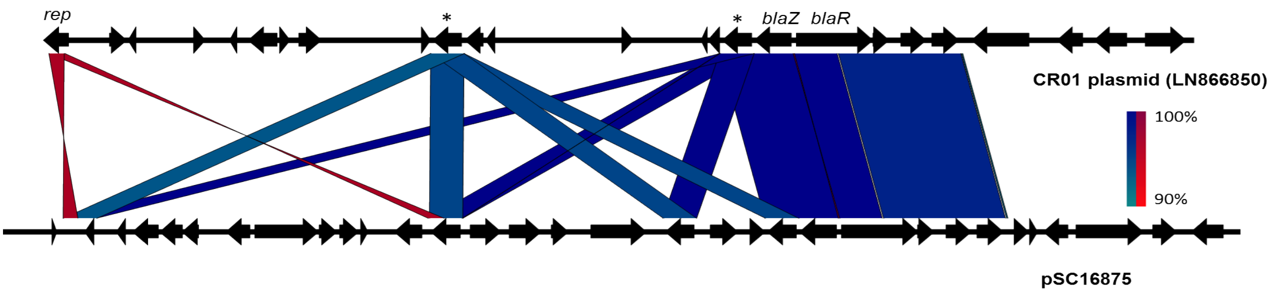

Supplementary Figure 3.

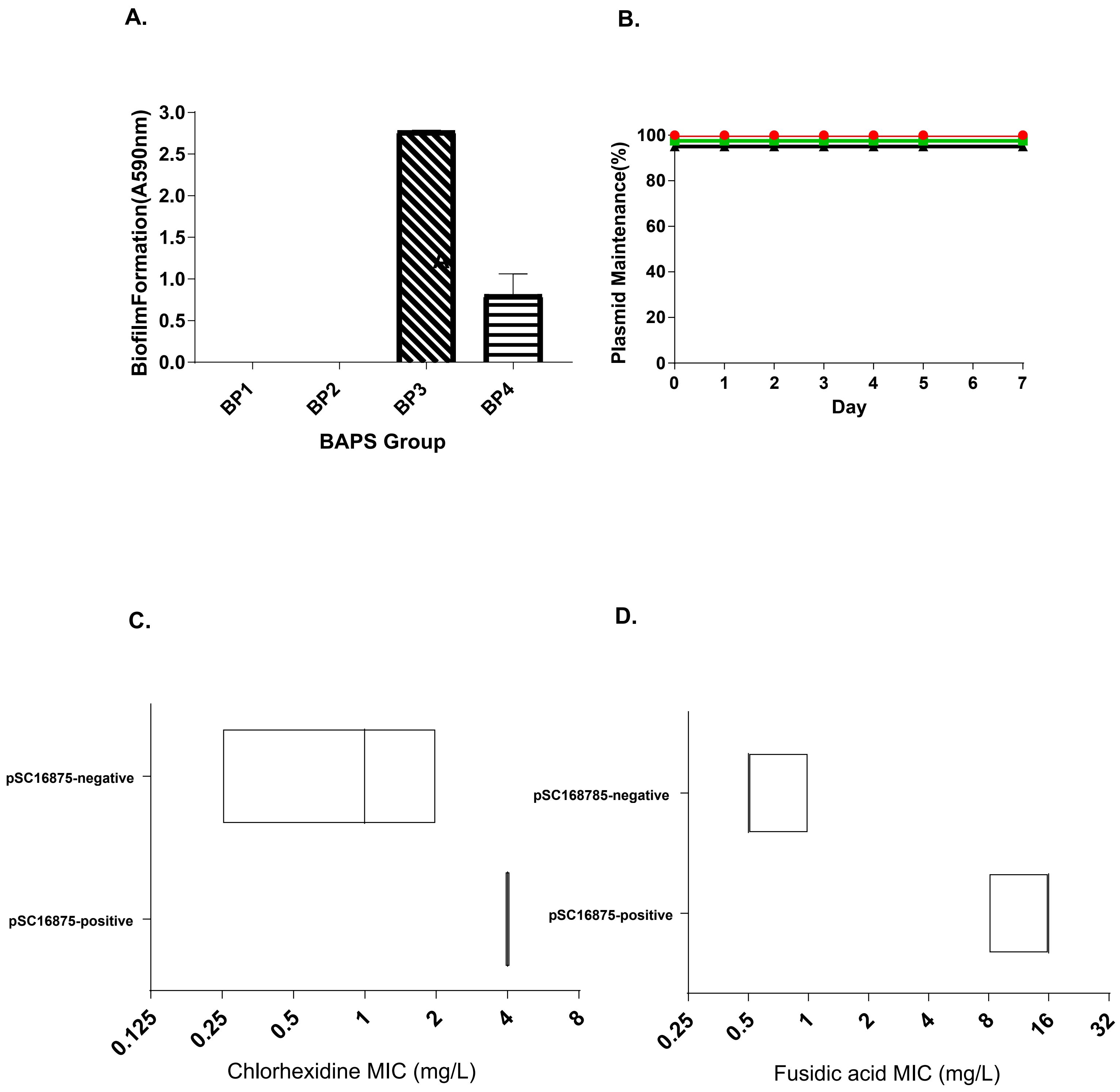

Supplement: Supplemental file 1 [file zac011187599s1.pdf]
